# Neonatal cause-of-death estimates for the early and late neonatal periods for 194 countries: 2000–2013

**DOI:** 10.2471/BLT.14.139790

**Published:** 2014-11-17

**Authors:** Shefali Oza, Joy E Lawn, Daniel R Hogan, Colin Mathers, Simon N Cousens

**Affiliations:** a MARCH, London School of Hygiene & Tropical Medicine, Keppel Street, London, WC1N 7HT, England.; b Department of Health Statistics and Information Systems, World Health Organization, Geneva, Switzerland.

## Abstract

**Objective:**

To estimate cause-of-death distributions in the early (0–6 days of age) and late (7–27 days of age) neonatal periods, for 194 countries between 2000 and 2013.

**Methods:**

For 65 countries with high-quality vital registration, we used each country’s observed early and late neonatal proportional cause distributions. For the remaining 129 countries, we used multinomial logistic models to estimate these distributions. For countries with low child mortality we used vital registration data as inputs and for countries with high child mortality we used neonatal cause-of-death distribution data from studies in similar settings. We applied cause-specific proportions to neonatal death estimates from the United Nations Inter-agency Group for Child Mortality Estimation, by country and year, to estimate cause-specific risks and numbers of deaths.

**Findings:**

Over time, neonatal deaths decreased for most causes. Of the 2.8 million neonatal deaths in 2013, 0.99 million deaths (uncertainty range: 0.70–1.31) were estimated to be caused by preterm birth complications, 0.64 million (uncertainty range: 0.46–0.84) by intrapartum complications and 0.43 million (uncertainty range: 0.22–0.66) by sepsis and other severe infections. Preterm birth (40.8%) and intrapartum complications (27.0%) accounted for most early neonatal deaths while infections caused nearly half of late neonatal deaths. Preterm birth complications were the leading cause of death in all regions of the world.

**Conclusion:**

The neonatal cause-of-death distribution differs between the early and late periods and varies with neonatal mortality rate level. To reduce neonatal deaths, effective interventions to address these causes must be incorporated into policy decisions.

## Résumé

**Objectif:**

Estimer les distributions des causes de décès dans les périodes néonatales précoces (âge compris entre 0 et 6 jours) et tardives (âge compris entre 7 et 27 jours), dans 194 pays entre 2000 et 2013.

**Méthodes:**

Pour 65 pays dotés d'un état civil de qualité supérieure, nous avons utilisé les distributions des causes de décès proportionnelles néonatales précoces et tardives, qui sont observées dans chaque pays. Pour les 129 pays restants, nous avons utilisé des modèles logistiques multinomiaux pour estimer ces distributions. Pour les pays à faible mortalité infantile, nous avons utilisé les informations de l'état civil comme données et, pour les pays à forte mortalité infantile, nous avons utilisé les données de distribution des causes de décès néonatales, qui ont été obtenues à partir d'études menées dans des sites similaires. Nous avons appliqué les proportions spécifiques aux causes aux estimations des décès néonatals du Groupe interinstitutions des Nations Unies pour l'estimation de la mortalité infantile par pays et par année pour estimer les risques spécifiques aux causes et le nombre de décès.

**Résultats:**

Au fil du temps, les décès néonatals ont diminué pour la majorité des causes. Sur les 2,8 millions de décès néonatals en 2013, 0,99 million (plage d'incertitude: 0,70–1,31) ont été estimés comme causés par des complications liées à une naissance prématurée, 0,64 million (plage d'incertitude: 0,46–0,84) par des complications au cours de l'accouchement et 0,43 million (plage d'incertitude: 0,22–0,66) par la septicémie ou d'autres infections graves. Les complications liées à la naissance prématurée (40,8%) et les complications au cours de l'accouchement (27%) représentaient la majorité des décès néonatals précoces, alors que les infections sont responsables de près de la moitié des décès néonatals tardifs. Les complications des naissances prématurées constituaient la principale cause de décès dans toutes les régions du monde.

**Conclusion:**

La distribution des causes de décès néonatales diffère entre les périodes précoces et tardives, et elle varie avec le niveau des taux de mortalité néonatale. Pour réduire les décès néonatals, des interventions efficaces permettant de traiter ces causes doivent être intégrées dans les décisions politiques.

## Resumen

**Objetivo:**

Estimar la distribución de las causas de muerte en los periodos neonatales tempranos (0–6 días de edad) y tardíos (7–27 días de edad) de 194 países entre 2000 y 2013.

**Métodos:**

Para 65 países con registros civiles de alta calidad, se utilizaron las distribuciones proporcionales de las causas neonatales tempranas y tardías observadas en cada país. Para los otros 129 países, se utilizaron modelos logísticos multinomiales a fin de calcular dichas distribuciones. Para los países con una mortalidad infantil baja se emplearon los datos de registros civiles como base, mientras que para los países con una mortalidad infantil alta se utilizaron los datos de distribución de causas de muerte neonatal de estudios realizados en contextos similares. Aplicamos las proporciones por causas específicas a las estimaciones de mortalidad neonatal del Grupo Interinstitucional de las Naciones Unidas para la Estimación de la Mortalidad Infantil, por país y año, para estimar los riesgos por causas específicas y el número de muertes.

**Resultados:**

A lo largo del tiempo, las muertes neonatales disminuyeron para la mayoría de las causas. De los 2,8 millones de muertes neonatales en 2013, se estimó que 0,99 millones de muertes (rango de incertidumbre: 0,70–1,31) se debían a complicaciones debidas a un parto prematuro, 0,64 millones (rango de incertidumbre: 0,46–0,84) por complicaciones durante el parto y 0,43 millones (rango de incertidumbre: 0,22–0,66) por sepsis y otras infecciones graves. Los partos prematuros (40,8 %) y las complicaciones durante el parto (27,0 %) representaron la mayor parte de las muertes neonatales precoces, mientras que las infecciones causaron casi la mitad de las muertes neonatales tardías. Las complicaciones en los partos prematuros fueron la causa principal de muerte en todas las regiones del mundo.

**Conclusión:**

La distribución de las causas de muerte neonatal difiere entre los periodos tempranos y tardíos y varía con el nivel de la tasa de mortalidad neonatal. Con objeto de reducir las muertes neonatales, es necesario incorporar intervenciones eficaces que traten estas causas en las decisiones políticas.

## ملخص

**الغرض:**

تقدير توزيعات أسباب الوفاة في فترات الولادة المبكرة (من سن 0 إلى 6 أيام) والمتأخرة (من سن 7 إلى 27 يوماً)، في 194 بلداً بين عامي 2000 و2013.

**الطريقة:**

بالنسبة للبلدان التي يوجد لديها تسجيل لشؤون الأحوال المدنية عالي الجودة التي يبلغ عددها 65 بلداً، قمنا باستخدام توزيعات الأسباب النسبية في فترات الولادة المبكرة والمتأخرة التي تم ملاحظتها في كل بلد. وبالنسبة للبلدان المتبقية البالغ عددها 129 بلداً، قمنا باستخدام النماذج اللوجيستية متعددة الحدود لتقدير هذه التوزيعات. وبالنسبة للبلدان التي تتميز بانخفاض معدل وفيات الأطفال، قمنا باستخدام بيانات تسجيل شؤون الأحوال المدنية في شكل مدخلات وبالنسبة للبلدان التي تتميز بارتفاع معدل وفيات الأطفال، قمنا باستخدام بيانات توزيع أسباب وفاة المواليد من الدراسات التي أجريت في بيئات مشابهة. وقمنا بتطبيق نسب محددة بالأسباب على تقديرات وفيات المواليد المستمدة من الفريق المشترك بين الوكالات المعني بتقدير وفيات الأطفال، حسب البلد والسنة، بغية تقدير المخاطر محددة الأسباب وأعداد حالات الوفاة.

**النتائج:**

بمرور الوقت، انخفضت حالات وفيات المواليد بالنسبة لمعظم الأسباب. ووفق التقديرات، كان سبب وفاة 0.99 مليون من أصل 2.8 مليون وفاة مواليد في عام 2013 (نطاق الاحتمال: من 0.70 إلى 1.31) هو مضاعفات الولادة قبل اكتمال مدة الحمل، وسبب وفاة 0.64 مليون (نطاق الاحتمال: من 0.46 إلى 0.84) هو المضاعفات أثناء الوضع وسبب وفاة 0.43 مليون (نطاق الاحتمال: من 0.22 إلى 0.66) هو الإنتان وغيرها من حالات العدوى الشديدة. وتعزى معظم وفيات المواليد في فترة الولادة المبكرة إلى مضاعفات الولادة قبل اكتمال مدة الحمل (40.8 %) والمضاعفات أثناء الوضع (27.0 %)، في حين تسببت حالات العدوى في نصف وفيات المواليد تقريباً في فترة الولادة المتأخرة. وكانت مضاعفات الولادة قبل اكتمال مدة الحمل السبب الرئيسي للوفاة في جميع أقاليم العالم.

**الاستنتاج:**

يختلف توزيع أسباب وفاة المواليد بين الفترتين المبكرة والمتأخرة ويتباين بتباين مستوى معدل وفيات المواليد. ولتقليل وفيات المواليد، يتعين إدراج تدخلات فعالة في القرارات المعنية بالسياسات بغية معالجة هذه الأسباب.

## 摘要

**目的:**

估计2000年至2013年194个国家新生儿早期（0–6日龄）和晚期（7–27日龄）死因分布。

**方法:**

对于65个具有高质量生命登记的国家，我们使用每个国家观察的早期和晚期新生儿比例原因分布。对于剩余的129个国家，我们使用多项逻辑回归模型来估计这些分布。对于儿童死亡率较低的国家，我们使用生命登记数据作为输入；对于儿童死亡率较高的国家，我们使用相似条件研究得到的新生儿死因分布数据。我们按国家和年份对联合国儿童死亡率估计机构间小组的新生儿死亡估计应用因别比率，以此估计各种原因的风险和死亡的数量。

**结果:**

随着时间的推移，大多数原因的新生儿死亡率有所降低。2013年，在280万新生儿死亡人数中，估计有99万例死因（不确定范围：0.70–1.31）为早产并发症，64万（不确定范围：0.46–0.84）为产时并发症，43万（不确定性范围：0.22–0.66）为败血症和其他严重的感染。早产（40.8%）和产时并发症（27.0%）是大多数早期新生儿的死因，而晚期新生儿死因将近半数为感染。早产并发症是世界所有地区死亡的主要原因。

**结论:**

早期和晚期新生儿死因分布不同，并随新生儿死亡率水平变化。要降低新生儿死亡，必须在决策中纳入对这些死因有效干预的措施。

## Резюме

**Цель:**

Провести оценку причин неонатальной смертности в раннем (0-6 дней после рождения) и позднем (7–27 дней) неонатальных периодах для 194 стран за 2000–2013 годы.

**Методы:**

Для 65 стран с высоким качеством регистрации актов гражданского состояния были использованы наблюдаемые в этих странах пропорциональные распределения причин смертности в раннем и позднем неонатальных периодах. Для остальных 129 стран были использованы мультиномиальные логистические модели для оценки этих распределений. Для стран с низкими показателями детской смертности в качестве входных данных использовались записи актов регистрации гражданского состояния, а для стран с высокой детской смертностью — данные распределений причин неонатальной смертности, указанные в исследованиях, проведенных в аналогичных условиях. Для оценки рисков в зависимости от причинного фактора и числа смертей были применены пропорции в зависимости от причинного фактора по конкретной стране и году к оценкам неонатальной смертности, полученным от Межведомственной группы ООН по оценке детской смертности.

**Результаты:**

С течением времени число неонатальных смертей по большинству причин снизилось. Согласно оценкам, из 2,8 млн. смертей новорожденных в 2013 году 990 000 смертей (область неопределенности: 0,70–1,31) было вызвано осложнениями, связанными с преждевременными родами, 640 000 смертей (область неопределенности: 0,46–0,84) произошло в связи с осложнениями во время родов, а 430 000 смертей (область неопределенности: 0,22–0,66) было вызвано сепсисом и другими тяжелыми инфекциями. На преждевременные роды (40,8%) и осложнения при родах (27,0%) приходилось большинство случаев ранней неонатальной смертности, в то время как причиной почти половины случаев поздней неонатальной смертности являлись инфекции. Осложнения при преждевременных родах были основной причиной младенческой смертности во всех регионах мира.

**Вывод:**

Распределение причин неонатальной смертности различно для раннего и позднего периодов и зависит от общего уровня неонатальной смертности. Для снижения неонатальной смертности государственная политика должна включать в себя эффективные меры по устранению этих причин.

## Introduction

In 2013, 2.8 million neonatal deaths occurred globally, most of which could have been prevented with optimal care.^1^ Although neonatal mortality is decreasing, the rate of reduction has been slower than that observed for under-5 mortality^2^^,^^3^ and neonatal deaths now constitute 44% of all deaths in children younger than 5 years.^1^ Slow progress in reducing neonatal deaths will contribute to many countries’ inability to meet the Millennium Development Goal 4 (MDG-4) target of reducing child mortality by two thirds between 1990 and 2015.^4^ To accelerate progress in reducing neonatal mortality, the *Every newborn: an action plan to end preventable deaths* was launched in June 2014^5^ to provide a strategy for implementing effective cause-specific interventions.

Understanding the neonatal cause-of-death distribution is important for identifying appropriate interventions and programme priorities. Information about the distribution should be as current and as locally derived as possible, and use cause categories that are programmatically relevant for decision-making. Moreover, separate cause-of-death estimates are required for the early (0–6 days of age) and late (7–27 days of age) neonatal periods since both biology and empirical data suggest that the cause-of-death distribution differs substantially between these periods.^6^^,^^7^ Around three-quarters of neonatal deaths occur during the early period^8^ and most interventions to prevent these deaths need to be delivered within a very short time frame.

High-quality vital registration data, by cause and age at death, provide the information needed to determine policies and priorities. Quality is determined by completeness of reporting and accuracy of cause-of-death coding.^9^^,^^10^ High-quality vital registration data are only available for one-third of the World Health Organization’s (WHO’s) Member States,^11^ which account for about 4% of neonatal deaths. For countries without high-quality vital registration data, statistical modelling is needed to estimate cause-of-death distributions.

Our first estimates of causes of neonatal deaths were published in 2005, for the year 2000.^12^ These estimates were obtained using data from high-quality vital registration systems and from studies in low-resource settings with high mortality in which high-quality vital registration data were lacking. Updates for 2008^13^ and 2010^14^ have since been published.

To aid policy-makers and programme managers, we have now estimated neonatal causes of death separately for the early and late neonatal periods, and added injuries as a distinct cause for low-mortality countries. The input data have also been updated and the modelling strategy has been modified, particularly for the split of neonatal infections between pneumonia and sepsis. We present global, regional, and national estimates of proportions, risks, and numbers of deaths for programmatically relevant neonatal causes of death by the early and late neonatal periods.

## Methods

**Fig. 1:**
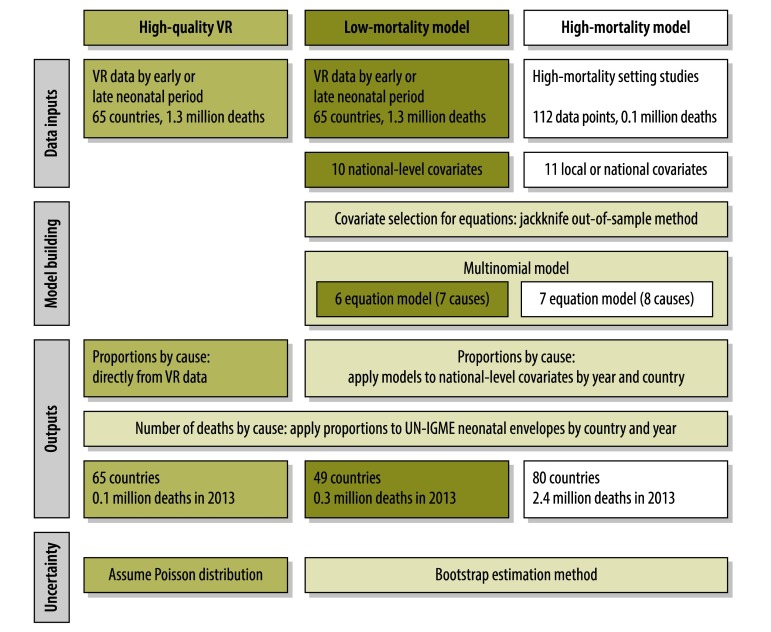
Flowchart of modelling methods to estimate country-specific distributions of neonatal cause of deaths

UN-IGME: United Nations Inter-agency Group for Child Mortality Estimation; VR: vital registration.

Notes: Details on the differences between this and previous modelling methods can be found in Appendix B (available from: http://arxiv.org/abs/1411.4021). Early neonatal period is defined as age 0–6 days and late neonatal period as age 7–27 days.

We divided WHO’s 194 Member States into three groups: group 1 contains countries with high-quality vital registration data, group 2 contains countries with poor-quality vital registration data and a low child mortality rate, and group 3 contains countries with poor-quality vital registration data and a high child mortality rate (Appendix A, available from: http://arxiv.org/abs/1411.4021). Different methods were used to estimate the proportional cause-of-death distributions for countries in each group (Fig. 1). In groups 1 and 2, we used seven categories for causes of death: complications of preterm birth, intrapartum complications, congenital disorders, pneumonia, sepsis and other severe infections (sepsis), injuries, and other causes. For group 3, we used eight categories: preterm birth, intrapartum complications, congenital disorders, pneumonia, diarrhoea, neonatal tetanus, sepsis, and other causes (Table 1). In Appendix B (available from: http://arxiv.org/abs/1411.4021), we detail the differences between these methods and those used for previous estimates.

**Table 1:** Case definitions for neonatal causes of death

Cause	Case definitions for neonatal causes of death	
	Used in VR^a^ and preferred in study data	Alternative definition accepted in study data
Preterm birth complications	– Specific complications of preterm birth, such as surfactant deficiency, intraventricular haemorrhage, necrotizing enterocolitis – Prematurity (< 34 weeks) at which level preterm complications occur for most babies – Neonatal death with birth weight < 2000 g for whom gestational age is unknown	– Prematurity – Very low birth weight
Intrapartum complications	– Neonatal encephalopathy with criteria suggestive of intrapartum events – Early neonatal death in a full term baby with no congenital malformations and a specific history of acute intrapartum insult or obstructed labour	– Birth asphyxia with Apgar-based definition but excluding preterm infants – Fits and/or coma in the first two days of life in a baby born at term – Acute intrapartum complications
Congenital disorders	– Major or lethal congenital abnormalities – Specific abnormality listed (e.g. neural tube defect, cardiac defect)	– Congenital abnormality or malformation
Sepsis	– Sepsis/septicaemia, meningitis, or neonatal infection	– Neonatal infection
Pneumonia	– Pneumonia or acute respiratory tract infection	– Pneumonia
Neonatal tetanus	– Tetanus	– Spasms and poor feeding in neonates older than 3 days
Diarrhoea	– Diarrhoea	– Diarrhoea
Injuries^b^	– Injuries (in VR data only)	– NA
Other	– Neonatal jaundice – Haemorrhagic disease of the newborn – Term baby dying due to in-utero growth restriction – Injuries (only searched for in study data)	– Author grouping of other causes (excluding unknown)

ICD: International Statistical Classification of Diseases and Related Health Problems; NA: not applicable; VR: vital registration.

^a^ Specific ICD codes for causes of death in the VR data are included in Appendix C (available from: http://arxiv.org/abs/1411.4021).

^b^ Only applied to vital registration data.

Notes: Neonatal period defined as age 0–27 days or first month after birth. Early neonatal period defined as age 0–6 days or first week after birth. Late neonatal period defined as age 7–27 days or weeks 2–4 after birth.

Source: Adapted from Lawn,^15^ which adapted from Wigglesworth.^16^ and Winbo et al.^17^

For group 1, the 65 countries with high-quality data, the proportional cause distribution from 2000–2013 was obtained directly from each country’s vital registration data.^18^ We mapped the reported causes of death to our cause categories (Appendix C, available from: http://arxiv.org/abs/1411.4021). We then generated a proportional cause distribution by dividing the number of deaths attributed to each cause by the total deaths. To create a full time series, we imputed the cause-specific proportions for years with missing vital registration data (Appendix D, available from: http://arxiv.org/abs/1411.4021).

For group 2, the 49 countries with poor-quality data and low child mortality, we estimated the distribution from 2000–2013 using a multi-cause (low-mortality) model with input data from high-quality vital registration countries, excluding imputed data.

For group 3, the 80 countries with poor-quality data and high child mortality, we estimated the distribution from 2000–2013 using a multi-cause (high-mortality) model. The input data consisted of neonatal cause-of-death distribution data from studies in high-mortality settings. We updated a previously developed database of neonatal cause-of-death studies^19^ by conducting an extensive literature review for relevant research published from January 2011 to May 2013 (Appendix E, available from: http://arxiv.org/abs/1411.4021). From each study, we extracted cause-of-death data and mapped these reported causes to our eight cause categories (Appendix F, available from: http://arxiv.org/abs/1411.4021). The data did not provide sufficient information on injuries for separate estimation. We recorded deaths separately by early or late neonatal period whenever possible.

For our models, we chose covariates that we believed might partly predict variation in the cause-of-death distribution across countries (Appendix G, available from: http://arxiv.org/abs/1411.4021). We obtained these covariates from WHO, the United Nations Children’s Fund, the United Nations Inter-agency Group for Child Mortality Estimation (UN-IGME) and the World Bank. We only used covariates for which national time series are publically available. We used national-level covariates as inputs to the low-mortality model since the input cause-of-death data are national. For the high-mortality model, local-level data were extracted from the studies whenever possible. When such covariate data were unavailable, we used subnational- or national-level covariate data instead. For the predictions, we applied the final model coefficients to national-level covariate data, with the exception of India, for which we used state-level data to produce state-level estimates. We applied the same rules for imputing missing covariate data as for the vital registration data (Appendix D). While most covariate values were within the ranges of the input data, a few prediction covariates had values substantially outside the input data range, especially in the low-mortality model (Appendix G). We capped the prediction covariate data to the input ranges in the analysis. However, a sensitivity analysis without these caps suggested that the decision to cap or not had minimal influence on the results (Appendix H, available from: http://arxiv.org/abs/1411.4021).

### Statistical modelling

All statistical analyses were done using Stata version 12 (StataCorp. LP, College Station, United States of America). We chose preterm birth as the baseline cause for the low-mortality model (the most common cause) and intrapartum complications as the baseline for the high-mortality model (reported in all studies). Our estimation process had two stages. First, we selected covariates. Then, we estimated the log-cause ratio (the log of the ratio of each of the other causes to the baseline cause) as a function of the selected covariates using a multinomial logistic regression model. For both the low- and high-mortality models, we ran separate models for the early and late neonatal periods. Thus, we fitted four models in total. Since not all studies in the high-mortality model reported deaths by period, we included a binary covariate for period in the models to be able to include studies reporting only causes of death across the whole neonatal period.

#### Covariate selection for models

For each of the four models, we used a jackknife procedure^14^ to select the set of covariates that minimized the out-of-sample prediction error for each log-cause ratio separately. First, we determined if the relationship between each covariate and log-cause ratio in the input data was best represented by a linear, quadratic or restricted cubic spline relationship. We did this by choosing the covariate relationship which yielded the smallest *χ^2^* value for the given log-cause ratio. We then selected the covariate with the smallest *χ^2^* as the first covariate in the model. Finally, to select a set of covariates for each non-baseline cause in each model, we added one covariate at a time, retaining it in the model only if *χ^2^* decreased and cycling through all the remaining covariates again until there was no decrease in the *χ^2^* value.

#### Multi-cause models

For the multi-cause models, we used the multinomial logistic regression to fit the data for all causes simultaneously. Each input observation received a weight inversely proportional to the square root of the total deaths contributed by that observation. This weighting is intermediate between giving equal weight to each death and equal weight to each study or country-year in the input data. We made assumptions about the cause category into which deaths from an unreported cause would have been assigned (Appendix I, available from: http://arxiv.org/abs/1411.4021). The coefficient values from the multinomial logistic regression models were applied to the country- and year-specific national level predictor covariates to estimate the proportional cause distribution for each modelled country from 2000–2013.

### Estimation

We used neonatal deaths and live births estimates produced by UN-IGME for each country from 2000–2013.^1^ The overall neonatal deaths from UN-IGME were divided into early and late neonatal deaths. For countries with high-quality vital registration, we took this split directly from the vital registration data. For the other countries, we assumed that 74% of neonatal deaths occurred during the early period and 26% in the late period based on previous work.^8^ We performed a sensitivity analysis in which we assumed the early proportion to be either 65% or 85% (Appendix J, available from: http://arxiv.org/abs/1411.4021). To determine the number of deaths by cause, by neonatal period, and by year for each country, we applied the cause-specific proportions to the period-specific UN-IGME neonatal deaths for each country and year. We then divided the estimated deaths by the relevant country-specific live births to obtain the risk per 1000 live births. We estimated cause-specific global and regional neonatal death totals by summing the results for the relevant countries.

#### Uncertainty estimation

For the modelled estimates, we generated uncertainty estimates by drawing 1000 bootstrap samples with replacement from the input data and re-running the multi-cause models to produce new proportional cause distributions. We took the 2.5 and 97.5 percentile values for each cause as the uncertainty bounds. For the high-quality vital registration data, we developed uncertainty estimates by assuming a Poisson distribution for the number of deaths.

## Results

**Fig. 2:**
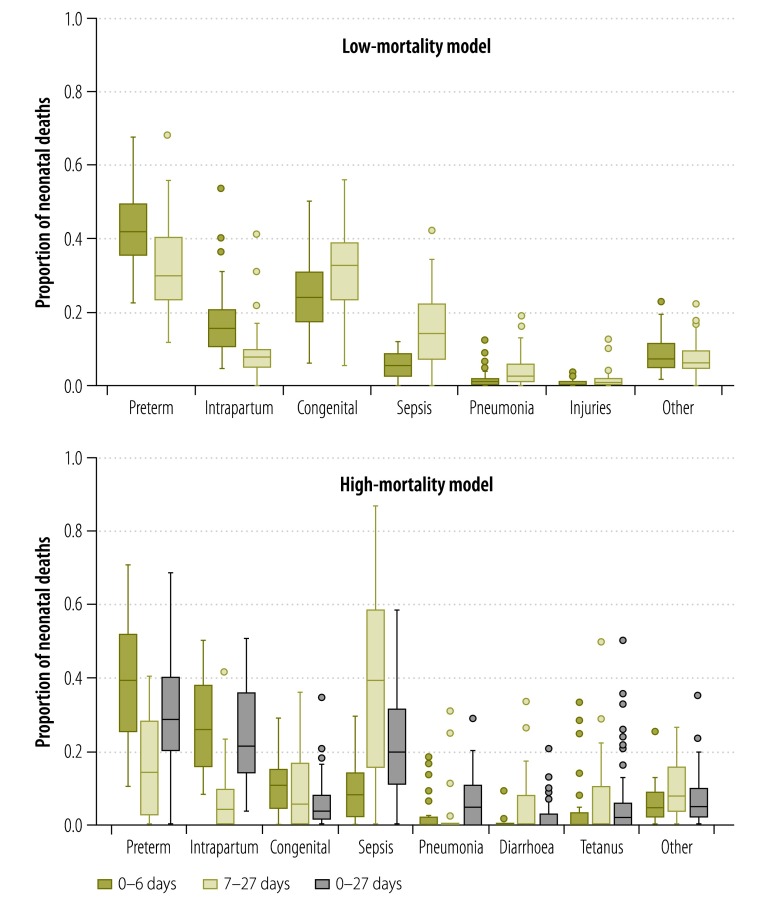
Proportional cause distribution of neonatal deaths by neonatal period for the input data in the low- and high-mortality models

Notes: The plot for the low-mortality model is for one observation per country by averaging across years. Early neonatal period is defined as age 0–6 days, late neonatal period as age 7–27 days and overall period as 0–27 days.

The high-quality vital registration input data set included 65 countries with 1 267 404 neonatal deaths and 665 country-years per neonatal period (Fig. 1). Of these deaths, 75.8% occurred in the early period. Preterm birth and congenital disorders’ causes were the most common causes during both neonatal periods (Fig. 2).

The high-mortality model input data set included 112 data points consisting of 98 222 deaths from 36 countries (Fig. 1). This includes the addition of nearly 4100 neonatal deaths from 15 new studies, representing 10 countries across five MDG regions (the countries in each region can be found in Appendix A), to the existing database. The overall data set had 31 observations for the early period, 18 for the late period, and 63 for the overall neonatal period. Seventy-eight observations had missing information on one or more cause, with pneumonia and diarrhoea being the causes most commonly unreported (Appendix I). Preterm birth and intrapartum complications were the most common causes of death during the early period, while infections (sepsis and pneumonia) dominated during the late period (Fig. 2).

### Statistical modelling

Covariate selection is presented in Appendix K (available from: http://arxiv.org/abs/1411.4021) and model regression coefficients are in Appendix L (available from: http://arxiv.org/abs/1411.4021).

#### Cause-specific deaths and risks

In 2013, the leading causes of neonatal death globally were preterm birth (35.7%), intrapartum complications (23.4%) and sepsis (15.6%), accounting for 2.1 million (uncertainty range: 1.4–2.8) of 2.8 million neonatal deaths (Table 2). Sensitivity analyses with high and low proportions of early deaths showed little differences for modelled countries (Appendix J).

**Table 2:** Global cause-specific numbers of neonatal deaths, proportions and risks in 2013

Cause	Early neonatal period			Late neonatal period			Total		
	No. of deaths^a^ (uncertainty range)	Proportion, %		No. of deaths^a^ (uncertainty range)	Proportion, %		No. of deaths^a^ (uncertainty range)	Proportion, %	Risk^b^
Preterm birth	834.8 (608.1–1083.5)	40.8		152.1 (91.0–229.0)	21.2		986.9 (699.1–1312.5)	35.7	7.2
Intrapartum complications	552.7 (407.6–711.4)	27.0		92.1 (54.8–133.4)	12.9		644.8 (462.4–844.7)	23.4	4.7
Congenital disorders	217.0 (140.9–325.9)	10.6		72.8 (42.5–124.5)	10.2		289.8 (183.3–450.4)	10.5	2.1
Sepsis	163.7 (62.4–271.6)	8.0		266.7 (156.5–393.2)	37.2		430.4 (218.9–664.8)	15.6	3.1
Pneumonia	98.9 (48.8–200.3)	4.8		37.6 (21.5–58.7)	5.2		136.4 (70.3–259.0)	4.9	1.0
Diarrhoea^c^	6.7 (0–57.4)	0.3		10.0 (3.2–25.6)	1.4		16.6 (3.2–83.0)	0.6	0.1
Tetanus^c^	21.1 (7.4–53.2)	1.0		27.1 (8.1–67.2)	3.8		48.2 (15.5–120.4)	1.7	0.3
Other^d^	149.9 (72.7–250.3)	7.3		57.9 (26.3–117.2)	8.1		207.8 (99.0–367.4)	7.5	1.5

^a^ In thousands.

^b^ Per 1000 live births.

^c^ Only estimated for the 80 high-mortality model countries.

^d^ Injuries are included in this category.

Note: Inconsistencies arise when percentages are added up due to rounding.

In the early period, preterm birth (40.8%) and intrapartum complications (27.0%) accounted for the majority of deaths while in the late neonatal period nearly half of all deaths occurred from infectious causes (47.6%; Table 2). The proportion of deaths from congenital disorders was relatively stable across the periods. Higher neonatal mortality rates and lower national income levels were associated with a higher proportion of deaths attributable to intrapartum complications and infectious causes (Appendix M, available from: http://arxiv.org/abs/1411.4021). The variation between the 10 MDG regions appears to reflect the differences in neonatal mortality rate between these regions. In low-mortality settings, injuries accounted for less than 1% of neonatal deaths, and this fraction increased slightly from the early to late period (Appendix M). See Appendix N (available from: http://arxiv.org/abs/1411.4021) for model-specific results, Appendix O (available from: http://arxiv.org/abs/1411.4021) for country-specific results and Appendix P (available from: http://arxiv.org/abs/1411.4021) for a comparison of results for China.

**Fig. 3:**
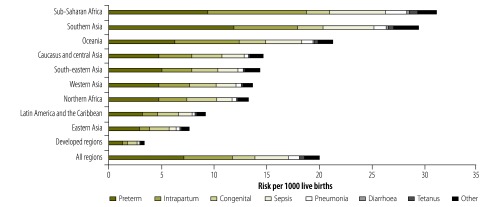
Cause-specific risk of neonatal death by Millennium Development Goal region in 2013

Note: The countries in each region are available from the United Nations^20^ and Appendix A (available from: http://arxiv.org/abs/1411.4021).

**Fig. 4:**
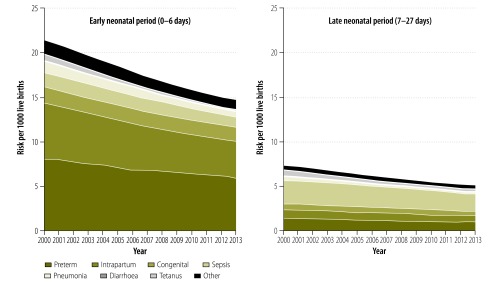
Global cause-specific risks of neonatal death for the early and late neonatal periods, 2000–2013

Globally, as the neonatal mortality rate has been decreasing over time, so have cause-specific risks. The risk of death from each cause is higher in high-mortality settings, even for causes that dominate proportionally in low-mortality settings. The risks of death due to preterm birth, intrapartum complications, and sepsis are 10, 36, and 34 times greater, respectively, in settings with more than 30 neonatal deaths per 1000 live births compared to settings with less than 5 neonatal deaths per 1000 live births. In every MDG region, preterm birth is the leading cause of neonatal death, with the highest risks in southern Asia (11.9 per 1000 live births) and sub-Saharan Africa (9.5 per 1000 live births; Fig. 3). The absolute risks of death due to preterm birth and intrapartum complications have been decreasing more in the early period than the late period (Fig. 4). Between 2000 and 2013, the largest absolute risk reduction in the overall neonatal period was estimated for intrapartum complications; dropping from 7.2 deaths (4.8–9.5) to 4.7 deaths (3.4–6.1) per 1000 live births. The largest relative decrease in risk was predicted for neonatal tetanus, which dropped by 73% (from 1.1 deaths [0.3–2.8] to 0.3 deaths [0.1–0.9] per 1000 live births) between 2000 and 2013. The smallest relative decrease in risk was predicted for congenital disorders (13% drop; from 2.4 deaths [1.4–4.1] to 2.1 deaths [1.3–3.3] per 1000 live births; Appendix Q, available from: http://arxiv.org/abs/1411.4021).

## Discussion

We developed comparable estimates of programmatically relevant causes of death in the early and late neonatal periods for 194 countries. The proportional neonatal cause distribution varied with several factors, including the age of death, the national neonatal mortality rate and over time. To reduce neonatal deaths, these variations must be understood and incorporated into decisions regarding the selection of appropriate interventions. With the launch of the *Every newborn: an action plan to end preventable deaths*, this is the time to tailor interventions to the individual circumstances of countries.

While the leading causes of neonatal death are the same for the early and late neonatal periods, the distributions of theses causes differ between the two periods. In the early period, preterm birth and intrapartum complications account for two-thirds of deaths while infections account for around 14% of deaths. In the late period, around a third of deaths are due to preterm birth or intrapartum complications while almost half are from infections.

Within each neonatal period, differences exist in the proportional cause distribution by level of neonatal mortality rate. Generally, neonatal mortality is closely linked to the level of care available to neonates. Settings with very low neonatal mortality tend to provide neonatal intensive care units, while areas with high neonatal mortality often cannot deliver simple interventions such as clean delivery kits, resuscitation equipment and antibiotics. We estimated that in low-mortality countries, congenital disorders caused more neonatal deaths than intrapartum complications or infections, while the opposite was true in high-mortality settings.

We used our model to predict trends in causes of death. Our model predicts that deaths due to intrapartum complications had the largest absolute risk reduction. The relative decrease in tetanus may be due to increases in clean deliveries, facility birth, cord care and tetanus toxoid vaccination, as well as contextual changes in maternal education and social norms.^21^ Additionally, a few countries in the low-mortality model eliminated neonatal tetanus after 2000. Since tetanus was not estimated in the low-mortality model, we may have underestimated the relative decline in risk.

The predicted risk of death due to preterm birth complications only represented a 25% relative decrease, despite the existence of cost-effective interventions such as antenatal corticosteroids and kangaroo mother care.^8^^,^^22^ In addition to prematurity, there is also evidence that babies that are small-for-gestational age are at higher risk of death.^23^

Our results are similar to previous estimates,^14^ with the exception that we estimate fewer pneumonia deaths than before. This is likely due to improvements to the estimation approach, namely the inclusion of additional studies that split pneumonia from sepsis and the inclusion of pneumonia directly in the multinomial model (Appendix B).

Although the quantity and quality of data have improved in recent years, data gaps still exist. We now have nearly 100 000 deaths and over 90 studies in the high-mortality model compared to less than  14 000 deaths and less than 60 studies for our first estimates.^12^ However, while we used the high-mortality model for 80 countries, the inputs only included data from 36 countries. Many of these studies were relatively small and few were nationally representative. We included data from only 13 sub-Saharan African countries, the region with the highest risk of neonatal death. Excluding a large South African data set, the studies from these countries contributed only 4000 deaths to our database.

As with all modelling exercises, our estimates should be viewed as an interim measure to help policy-makers, particularly in settings with little or no available data. It is important to distinguish estimates from data and to recognize that not all estimates are equally robust. We used UN-IGME estimates of the neonatal mortality rate and total number of neonatal deaths in each country. The UN-IGME estimates of all-cause mortality in each country are derived from data for that country and therefore can be said to track mortality in each country. For most countries, our cause-specific estimates are not based on data from that country, but from a model bringing together data from many countries. The model then predicts the cause-of-death distribution and changes in this distribution in individual countries, based on country-specific covariate values. Some countries contribute little or no input data to the modelling process. For example, only 24 deaths in our input data came from Nigeria, one of the most populous countries. For the majority of countries, our estimates should therefore not be interpreted as tracking changes in causes of death, but rather as predictions of what might be occurring in countries. To track changes of specific causes of death requires each country to collect representative and consistent data on causes of death on a continuing basis.

Some countries, like South Africa, have made rapid improvements to their vital registration systems.^24^ We made a set of proposals for improving the reporting of births and neonatal deaths that we reported in the* Lancet*.^3^ As collection of data improves, estimates should be replaced by reliable local cause-of-death data.

The validity of our estimates relies on the quality of the input and prediction data, and on our modelling techniques. Quality is of concern for verbal autopsy studies, in which the reported cause distributions depend on the case definitions and causal hierarchies used to attribute deaths.^10^ Accurate cause attribution using verbal autopsy will always be problematic for causes that are difficult to distinguish – such as sepsis and pneumonia – or difficult to identify – such as internal congenital abnormalities. The potential lack of comparability between different verbal autopsy studies can affect the ability of our model to predict variation between settings. These problems of accuracy and comparability can be partially mitigated by following standard methods when conducting verbal autopsy studies.^25^ Additionally, regression-based models depend on the relationship between outcome variables and covariates, which should ideally come from the same population and time period. While we sought to include as much local covariate information as possible for the input studies, 52% of the total covariate information came from national data instead of from state or local data. Finally, when re-classifying reported verbal autopsy causes of death (Table 1), we had to make choices, for example placing deaths reported as being due to very low birth weight into the preterm birth complication category. This may introduce a degree of misclassification as some very low birth weight deaths may be attributable to congenital abnormalities. We made choices that we believed would introduce the least misclassification, but until verbal autopsy methods improve, this will continue to be a challenge. Similar issues exist in International Statistical Classification of Diseases and Related Health Problems (ICD) coding, but are more common in verbal autopsy studies because of the limited and lower quality of information collected.

Even high-quality vital registration data can have problems. ICD-10 codes are not ideal for neonatal causes because several programmatically relevant causes are relegated to the often-unused fourth digit in the codes. Codes for the upcoming ICD-11 revision are currently being drafted, providing the opportunity to develop more appropriate, clinically relevant coding for neonatal causes. Additionally, ICD coding practices can vary between and even within countries and over time.^26^^,^^27^ Such variations reduce our model’s ability to predict true variation in causes of death. Other issues in coding include differences in relegating certain causes to non-specific or ill-defined cause categories and the assumption inherent in our exclusion of such codes that the deaths attributed to them are a random sample of all deaths. Finally, the availability and quality of vital registration data in a country may change over time, especially in countries with newly emerging surveillance systems. Developing consistent time trend estimates given such changes remains a challenge.

We used multinomial models, which ensure that the sum of cause-specific deaths equals total deaths. Single-cause models require post-hoc adjustments to retain this property, and there may be limited information on which to base such adjustments. A concern for both types of models is the attribution of death to a single cause. This does not allow for co-morbidities, which are a frequent occurrence in neonatal deaths, and is therefore a simplification of the true situation. This in turn may affect the potential impact of interventions.

Given the considerable variations in health systems and contextual factors within individual countries, subnational neonatal cause-of-death estimates are needed and should be a target for future estimation exercises and data collection. National-level estimates aim to ascertain the average causal distribution for a country, which can help guide national priorities, but may mask subnational variation. Some countries are beginning to collect information for subnational estimates. For example, our national estimates for India were produced by aggregating state-level estimates. We also wish to further differentiate causes within the current broad categories such as congenital disorders. However, such differentiation may only be possible for vital registration-based models, since verbal autopsy-based data generally lack the needed information for such differentiation. Finally, we believe that the production of estimates should be transparent. Therefore, the datasets and Stata code we used for this analysis are available on the WHO Global Health Observatory website.^18^

Reducing neonatal deaths will be essential to achieve the unfinished MDG-4 agenda after 2015.^28^ The *Every newborn: an action plan to end preventable deaths*, which calls for all countries to reduce their neonatal mortality rates to 10 or fewer deaths per 1000 live births by 2035, provides a push for ending preventable newborn deaths.^5^
